# Gene regulation in response to host sex and infection route in *Brugia pahangi* with new genome annotation

**DOI:** 10.1093/g3journal/jkag073

**Published:** 2026-04-15

**Authors:** Christopher I Holt, Robin E Bromley, Benjamin C Sparklin, John Mattick, Jeremy M Foster, Michelle L Michalski, Julie C Dunning Hotopp

**Affiliations:** Institute for Genome Sciences, University of Maryland School of Medicine, Baltimore, MD 21201, United States; Institute for Genome Sciences, University of Maryland School of Medicine, Baltimore, MD 21201, United States; Institute for Genome Sciences, University of Maryland School of Medicine, Baltimore, MD 21201, United States; Institute for Genome Sciences, University of Maryland School of Medicine, Baltimore, MD 21201, United States; Biochemistry and Microbiology Division, New England Biolabs, Ipswich, MA 01938, United States; Department of Biology, University of Wisconsin Oshkosh, Oshkosh, WI 54901, United States; Institute for Genome Sciences, University of Maryland School of Medicine, Baltimore, MD 21201, United States; Department of Microbiology and Immunology, University of Maryland School of Medicine, Baltimore, MD 21201, United States; Greenebaum Cancer Center, University of Maryland School of Medicine, Baltimore, MD 21201, United States

**Keywords:** filariasis, *Brugia pahangi*, host sex effect, infection route, transcriptomics

## Abstract

Male rodents are almost exclusively used for experimental maintenance of filarial nematodes because they have higher susceptibility to parasitism than female rodents. This study investigates the effects that rodent host sex has on the worm transcriptome for subcutaneous (SQ) infections and intraperitoneal (IP) infections. Significantly more adult worms were recovered from IP-infected gerbils (median = 78 worms/infected animal) relative to SQ-infected gerbils (median = 2 worms/infected animal). Microfilaria production in male gerbils infected via the SQ route (mean = 6.62 microfilariae (mf)/20 µL, median = 5.65 mf/20 µL) was significantly greater than in female gerbils (mean = 0.43 mf/20 µL, median = 0 mf/20 µL). Male gerbils were also significantly more susceptible to SQ infection than females with a prevalence of 90% (*n* = 20) in males vs 27% (*n* = 29) in female gerbils. There was no significant difference in the number of adult worms recovered between male and female gerbils for either infection route. No statistically significant gene expression differences were observed in comparisons of worms of the same life stage/sex from male and female gerbils. In subcutaneously infected gerbils, the identification of differentially expressed genes was likely precluded by the markedly different transcript profiles between replicates. However, the transcriptional profiles for IP-infected gerbils were homogenous suggesting that the host sex does not alter nematode gene expression in the life stages examined from the IP model. The lack of impact of host sex on the transcriptome of worms isolated from IP-infected gerbils reinforces the use of male gerbils as the primary rearing host for these parasites. Genome annotation of *B. pahangi* is reported as it was required for this differential expression analysis.

## Introduction

Lymphatic filariasis is a mosquito-borne neglected tropical disease that is estimated to affect 67 to 120 million people across the equatorial regions of Latin America, Africa, and Southeast Asia (Taylor et al. 2010; Cano et al. 2014; Dickson et al. 2017; World Health Organization 2020). In humans, this disease is caused by 3 filarial nematodes: *Wuchereria bancrofti* (∼90% of cases), *Brugia malayi* (∼10% of cases), and *Brugia timori* (<1% of cases) (Fang and Zhang 2019; World Health Organization 2020). Bancroftian or Brugian filariasis can lead to the development of a lymphedema, usually caused by inflammatory responses against dead worms that lead to the retention of fluid and inflammation of the lymphatics in the arm, leg, or groin, manifesting as elephantiasis in severe cases (Babu and Nutman 2012; Liao and von der Weid 2015).

Men are disproportionately afflicted with lymphatic filariasis (Brabin 1990b; Pani et al. 1991). In 43 out of 53 epidemiological studies, women had a lower mean prevalence of lymphatic filarial infection compared with men (Brabin 1990b). Men represent 10% more cases of Bancroftian and 25% more cases of Brugian filariasis (World Health Organization 1994). In Malaysia, men had higher *B. malayi* microfilaremias compared with women, although women made up more of elephantiasis cases in certain situations (Wilson 1961). Male-biased parasitism is common in mammals and birds, and it has been attributed to sex-specific hormonal and behavioral differences (Bundy 1988). Differences in infection susceptibility have also been documented in onchocerciasis (Brabin 1990a), which is caused by a different filarial nematode, *Onchocerca volvulus*.

Sex-specific infection differences are not limited to filarial nematodes. Men comprise a higher percentage of infected individuals and have a differing immune response compared with women in both *Schistosoma* and *Leishmania* infections (Mugono et al. 2014; Dupnik et al. 2019; Ayabina et al. 2021; Bea et al. 2025; Honecker et al. 2026). Sociological and environmental factors contribute to this difference in infection rates, but sex is a factor (Mugono et al. 2014; Ayabina et al. 2021). With Schistosomiasis, environmental and sociological factors include a higher percentage of men taking part in water-adjacent activities such as swimming, farming, and fishing, with infection rates increasing in women when they take up these roles. In *Leishmania infantum* infections, male hosts develop larger lesions, have higher parasite burdens, and have a lower IFN-1 response, highlighting sex-specific differences in the immune response (Bea et al. 2025). Overall, there are sex-specific differences in the infection rates and immune response of both human and rodent hosts (Honecker et al. 2026).

Sex bias has also been observed in filarial nematode laboratory models, including in *B. malayi* and the related *Brugia pahangi*, which is a parasite of only nonhuman mammals. Male gerbils and male BALB/c mice are infected by *B. pahangi* more readily than their female counterparts (Ash 1971; Ah and Thompson 1973; Wesley 1973; Nakanishi et al. 1989) as also seen with male SCID mice and *B. malayi* (Rajan et al. 1994). This sex bias is observed in both subcutaneous (SQ) (Ash 1971; Ah and Thompson 1973; Wesley 1973) and intraperitoneal (IP) infection routes (Ah and Thompson 1973; Nakanishi et al. 1989; Rajan et al. 1994). Male gerbils (*Meriones unguiculatus*) subcutaneously inoculated in the groin with *B. pahangi* larvae have a greater susceptibility to infection and have 3 times as many adult worms recovered relative to females (Wesley 1973). Removal of the spleen or ovaries does not change the results in female gerbils (Wesley 1973), but treatment with testosterone (as testosterone propionate) or androgen (as testosterone propionate and cholesterol) increases the worm yield (Wesley 1973). Castration of male gerbils resulted in a lower number of worms removed from the gerbil and a reduced number of gerbils with detectable infection (Wesley 1973). Castrated males treated with testosterone have similar numbers of worms recovered from the gerbil and patent infections as intact males that do not receive hormone treatment (Wesley 1973). This suggests that the presence of testosterone plays an important role in host sex differences of filarial infection.

Two types of infection routes are used in the laboratory to rear both *B. malayi* and *B. pahangi*: IP infection and SQ infection (Ash and Riley 1970a, 1970b; McCall et al. 1973). IP infection involves injecting larvae into the peritoneal cavity of the mammalian host (McCall et al. 1973; Mutafchiev et al. 2014). By contrast, the SQ infection method involves injecting the larvae into the SQ layer, usually near the groin (Ash and Riley 1970a, 1970b). The IP route provides more worms recovered per gerbil and a shorter time frame to develop a patent infection (McCall et al. 1973), which is when an infection is detectable in the host. Adult worms and microfilariae in IP-infected gerbils are recovered almost entirely from the peritoneal cavity, whereas adults in SQ-infected gerbils are located in the lymphatics and shed microfilariae into the peripheral blood (Ah and Thompson 1973; McCall et al. 1973). Due to the ease of worm recovery, the IP infection route is the preferred method of rearing nematodes for laboratory studies that require high worm recovery.

Collectively, this means that most research studies use *B. malayi* or *B. pahangi* from IP-infected male gerbils (eg Choi et al. 2011; Ballesteros et al. 2016a, 2016b; Chung et al. 2019; Maclean et al. 2019; Voronin et al. 2019; Foster et al. 2020; Quek et al. 2022; Hegde et al. 2024)). Therefore, we sought to establish if there are transcriptional differences in filarial nematodes recovered from male and female gerbils and to examine the transcriptional differences between worms recovered from IP and SQ infections. We use *B. pahangi*, which typically provides a more robust infection in gerbils than *B. malayi* (Ash 1973).

## Materials and methods

### Study design, sample preparation, and statistical analyses

Mongolian gerbils (*Meriones unguiculatus*) were injected with *B. pahangi* larvae with 7 male gerbils and 7 female gerbils injected with 400 larvae into the IP cavity (IP infection) and 22 male gerbils and 32 female gerbils injected subcutaneously with 150 larvae (SQ infection). Five gerbils had to be removed from consideration in this study (Supplementary Table 1). Infection status in the IP-infected gerbils was established by the presence of worms at necropsy. Infection status in the SQ-infected gerbils was established by a blood draw at least 120 d postinfection to confirm the presence of microfilariae. Using this criteria, 18 male and 8 female gerbils developed successful SQ infection, while 21 SQ-infected female gerbils and 2 infected male gerbils did not develop infection. Out of the successfully infected gerbils, worms from 3 IP-infected male gerbils, 3 IP-infected female gerbils, 3 SQ-infected male gerbils and 3 SQ-infected female gerbils were selected for sequencing. To reduce the potential of batch effects, we aimed to keep litter mates consistent. However, this was not possible for the SQ-infected gerbils due to the low numbers of worms recovered. All animal care and protocols were compiled by the University of Wisconsin Oshkosh and approved by the University of Wisconsin Oshkosh IACUC. *T*-tests were performed in R v4.2.1 using stat_compare_means from ggpubr v0.4.0 and *χ*² analyses were performed in R v4.2.1 using stats v4.2.1. Power calculations for *t*-tests of means were performed using the R package pwr v1.3-0 (Champely 2020) with the options of a 1 sample *t*-test and the default option of a 2-sided alternative hypothesis.

### RNA-seq library preparation and sequencing

Two shipments of liquid nitrogen flash-frozen worms were shipped on dry ice from Oshkosh, WI, to Baltimore, MD, arriving on 25 March 2021, and 13 April 2021, where RNA was isolated from the adult male worms, adult female worms, and microfilariae using the NEB Biolabs Monarch Total RNA Miniprep kit (T2010) (New England Biolabs, Ipswich, MA) after physical disruption with a micropestle in liquid nitrogen. RNA samples were prioritized for library construction by balancing the selection of litter-matched samples with the selection of samples with the highest quantity and quality RNA. Sequencing libraries were constructed and sequenced by Maryland Genomics at the University of Maryland School of Medicine in Baltimore, MD, USA. Briefly, the isolated RNA underwent poly(A)-enrichment with the NEBNext Poly(A) mRNA magnetic isolation module. Libraries were constructed using the NEBNext Ultra II Directional RNA for Library Prep kit with PCR amplification steps using primers containing a 7-nt index sequence. Size selection and cDNA purification between enzymatic reactions were performed with the SPRIselect reagent (Beckman Coulter Genomics, Danvers, MA, USA). Libraries were evaluated using the GX touch capillary electrophoresis system (Perkin Elmer, Waltham, MA, USA). In a few cases when construction of key sequencing libraries failed, we selected new sets of litter-matched RNA samples in an effort to have the best data from litter-matched samples. The final selection of libraries was sequenced on the NovaSeq6000 with the S4 Reagent Kit v1.5 for 200 cycles. The *Wolbachia* endosymbiont of *B. pahangi* was not investigated in this study.

### Oxford Nanopore Technologies direct RNA sequencing of adult male and adult female *B. pahangi* for annotation

Live *B. pahangi* FR3 strain were isolated from 2 male gerbils infected in the IP cavity with ∼400 *B. pahangi* L3 larvae. The first gerbil was infected when 106 d old and was sacrificed 346 d postinfection to obtain 47 adult female worms and 55 male worms. The second gerbil was 135 d old and was sacrificed 464 d postinfection to obtain 26 adult female and 30 adult male worms. The worms were shipped in sterile filtered culture media consisting of RPMI 1640 (Thermo Fisher, Waltham, MA, USA), supplemented to a final concentration of 10% fetal bovine serum (Thermo Fisher, Waltham, MA, USA), 50 units/mL of penicillin (Thermo Fisher, Waltham, MA, USA), 50 µg/mL of streptomycin (Thermo Fisher, Waltham, MA, USA), and 40 µg/mL gentamicin (Sigma-Aldrich, Saint Louis, MO, USA). The worms were acclimated overnight at 37 °C and 5% CO_2_, in the same media they arrived in, before being flash frozen and physically disrupted with a micropestle in liquid nitrogen. RNA was isolated from a pool of all 73 females using the NEB Monarch Total RNA Miniprep kit (T2010). RNA was separately isolated from the pool of 55 male worms from the first gerbil using the NEB Monarch Total RNA Miniprep kit (T2010). The 23 ng *B. pahangi* female RNA and 5 ng *B. pahangi* male RNA were used separately to prepare libraries using the SQK-RN002 Direct RNA-Sequencing Kit and sequenced on an ONT MinION using the FLO-MIN106D R9 flow cell. Fast5 files from the 2 ONT sequencing runs were basecalled using Guppy v6.4.2, using the rna_r9.4.1_70bps_hac.cfg config file and a min_qscore of 7 (Guppy Basecalling Software).

### Illumina sequencing of cDNA for annotation

Aliquots of RNA from pools of *B. pahangi* L3 (NR-42499), pools of adult males (NR-42500), and pools of microfilariae (NR-48664) were acquired from FR3 through BEI Resources (Michalski et al. 2011) that contained 0.5 to 2 µg of RNA in TE buffer. These nematodes were reared in IP-infected male gerbils. The isolated RNA underwent poly(A)-enrichment with the NEBNext Poly(A) mRNA magnetic isolation module. Libraries were constructed using the NEBNext Ultra II Directional RNA for Library Prep kit with PCR amplification steps using primers containing a 7-nt index sequence. Size selection and cDNA purification between enzymatic reactions were performed with the SPRIselect reagent (Beckman Coulter Genomics, Danvers, MA, USA). Libraries were evaluated using the GX touch capillary electrophoresis system (Perkin Elmer, Waltham, MA) and sequenced on an Illumina HiSeq 4000. All Illumina FASTQ files underwent trimming with trimmomatic v0.38, using the TruSeq3-PE-2.fa adapter. Illumina fastq files were inspected with FASTQC before and after trimming (Andrews).

### Genome annotation

The *B. pahangi* reference genome underwent softmasking using RepeatMasker v4.0.7 and a RepeatModeler v1.0.11 database built using the NCBI engine (Smit and Hublet 2008–2015; Smit et al. 2015). ONT basecalled FASTQ files were aligned to the reference genome using minimap2 v2.17 with options -ax splice -uf -k14 (Lebov et al. 2020; Li 2021). Illumina reads were aligned to the reference genome index using hisat2 v2.1.0, with options –rna-strandness RF and –max-intronlen 5000 (Bolger et al. 2014; Kim et al. 2019; Mattick et al. 2020). Samtools v1.9 was used to sort and index the bam files (Danecek et al. 2021). The bam files from the Illumina data generated for the differential expression analysis underwent deduplication using Picard MarkDuplicates v2.25.3 (java version 1.8.0_17) (Broad Institute 2019). The resulting 14 bam files (Supplementary Table 2) were provided to BRAKER v2.1.6, along with the softmasked genome file, for structural annotation using Augustus v3.4.0 and Genemark ES/ET v4.62 (Stanke and Morgenstern 2005; Stanke et al. 2006, 2008; Li et al. 2009; Barnett et al. 2011; Lomsadze et al. 2014; Buchfink et al. 2015; Hoff et al. 2016, 2019; Mattick et al. 2020; Bruna et al. 2021). A protein only structural annotation was also generated with BRAKER v2.1.6 using the metazoan protein fasta file from OrthoDB v10 and ProtHint v2.6.0 (Altschul et al. 1990; Lomsadze et al. 2005; Stanke and Morgenstern 2005; Stanke et al. 2006, 2008; Gotoh 2008, 2014; Camacho et al. 2009; Iwata and Gotoh 2012; Buchfink et al. 2015; Hoff et al. 2016, 2019; Kriventseva et al. 2019; Bruna et al. 2020, 2021; Mattick et al. 2020). A combined GTF was generated using TSEBRA v1.0.3 (Gabriel et al. 2021) and formatted into a GFF3 file using custom R v4.1.2 scripts. A polypeptide fasta was generated using gffread v0.12.7. (Pertea and Pertea 2020). Functional evidence was generated using HMMER/Hmmscan, RNAmmer v1.2, tRNAscan-SE v2.0.3, TMHMM v2.0, and Rapsearch2 (Lowe and Eddy 1997; Krogh et al. 2001; Lagesen et al. 2007; Zhao et al. 2012). The evidence was evaluated and applied to the gene models using BioCode v0.10.0 and Attributor, resulting in a functionally annotated GFF3 file (Josh Orvis; J Orvis). Interproscan v5.56-89.0 was used to assign interpro (IPR) and gene ontology (GO) terms to each gene model (Jones et al. 2014).

### RNA-seq differential expression analysis

For the differential expression samples (Supplementary Table 3), the FASTQ files underwent a quality check using fastqc v0.11.9 (Andrews). The FASTQ files were then trimmed using trimmomatic v0.38 (java version 1.8.0_171) (Bolger et al. 2014) with the TruSeq3-PE-2.fa adapter and aligned to the *B. pahangi* reference genome (Mattick et al. 2020) using hisat2 v2.2.1 (Kim et al. 2019), with options –rna-strandness RF and –max-intronlen 5000. Samtools v1.9 was used to sort and index the bam files (Danecek et al. 2021). All bam files underwent deduplication using Picard MarkDuplicates v2.25.3 (java version 1.8.0_171) (Broad Institute 2019). For analyses where SQ files were merged, the merging of SQ bam files was performed with samtools v1.20 (Danecek et al. 2021) followed by Picard DownsampleSam v2.25.3 (java version 1.8.0_171) (Broad Institute 2019) to downsample the merged SQ bam files to 100 million reads. Once aligned and sorted by position, read counts were generated using HTSeq v0.12.4 from python 3.8.2 on gene features using union mode, with subsequent filtering to remove rRNAs and tRNAs (Anders et al. 2015). Only genes with features annotated as mRNA were considered. To ensure sequencing saturation, a rarefaction curve was generated using the R package vegan v2.5.7 (Dixon 2003; Oksanen et al. 2022). All samples reached acceptable saturation levels except for the SQ microfilariae, which were removed from the analysis. A dendrogram of the samples using *z*-score-normalized log_2_(TPM) values of differentially expressed genes was generated using pvclust v2.2-0 (Suzuki et al. 2019) and 1,000 bootstrap replications and default arguments for method.dist and method.hclust.

To assess the number of mapped reads to each chromosome, the trimmed reads were realigned to a composite reference file that included the *B. pahangi* nuclear, mitochondrial, and *Wolbachia* genomes and the bam files were deduplicated. Using the alignment method described above, the trimmed reads were also aligned to the gerbil reference genome (GCF_030254825). Samtools v1.20 idxstats (Danecek et al. 2021) was used to summarize the number of mapped reads in both cases.

Differential expression was determined using R v4.2.1 (R Core Team (2020)) and edgeR v3.30.3 (Robinson et al. 2010) and respective pairwise comparison matrices were made using limma v3.44.3 (Ritchie et al. 2015). Using edgeR, counts were normalized, and genes were filtered so that genes with at least a cpm value of 5 in 3 or more samples were kept. The common, tagwise, and trend dispersions were estimated for each sample and fitted to a generalized linear model. A quasi-likelihood *F*-test was used to identify differentially expressed genes, which were then filtered based on an FDR < 0.05, after Benjamini-Hochberg correction. Differentially expressed genes were clustered using WGCNA v1.71 (Langfelder and Horvath 2008).

The package tidyverse v1.3.2 (Wickham et al. 2019) was used for data processing, the package ggdendro v0.1.23 (de Vries and Ripley 2022) was used to acquire the order of the pvclust dendrogram, and the package devtools (Wickham et al. 2022) was used to download the heatmap.3 function. Interproscan v5.56-89.0 was used to assign IPR and GO terms to each gene model (Jones et al. 2014).

### Updated *B. malayi* functional terms

A differential expression analysis was undertaken to compare adult male, adult female, and mature microfilariae samples (Chung et al. 2019) using the read counts calculated in a previously published *B. malayi* transcriptomics meta-analysis (Holt and Dunning Hotopp 2024). Differentially expressed genes were identified with a quasi-likelihood *F*-test using R v4.2.1 (R Core Team (2020)) and edgeR v3.30.3 (Robinson et al. 2010) with the same cpm cutoff and a generalized linear model and clustered using WGCNA v1.71 (Langfelder and Horvath 2008), as described above. A protein fasta file was generated from the gff annotationf ile and the B. malayi WS276 genome reference downloaded from Wormbase Harris et al. 2020 and interproscan v5.56-89.0 (Jones et al. 2014) was used to assign the IPR and GO terms to each gene.

## Results

### Host sex and infection route effects on infection in gerbils

Gerbils were infected with third stage *B. pahangi* larvae using established FR3 protocols (Michalski et al. 2011) without alteration for either SQ or IP infection. These protocols are the current gold standard, reflecting what is considered rigorous for that infection mode, what is most frequently going to be used in studies, and what is an appropriate infection load for the animals. All *B. pahangi* larvae for experimental infections were isolated from mosquitos the same day as gerbil infection.

**Fig. 1.**
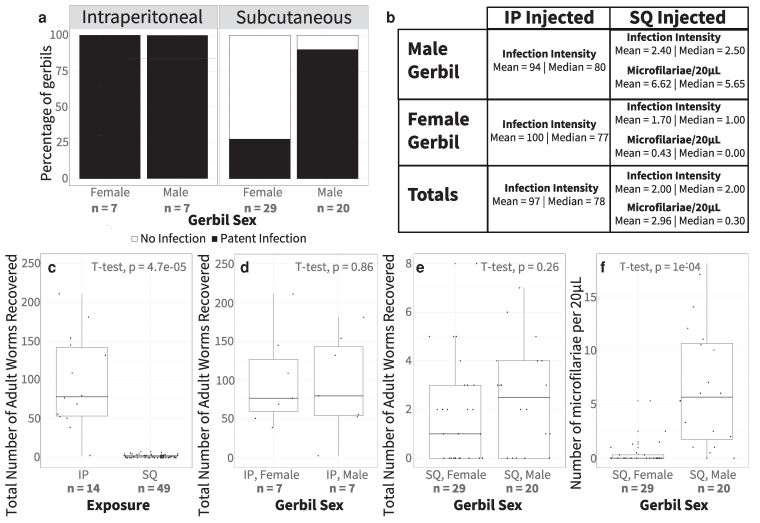
Study design and infection results. a) For the IP infection, all 7 of the female gerbils and all 7 of the male gerbils were successfully infected. For the SQ infection route, 18/20 (90%) male gerbils developed a successful infection while only 8/29 (28%) female gerbils developed a successful infection. Infection success rate is different for male and female gerbils using the SQ infection route, but not the IP infection route. b) Adult worm recovery and microfilariae levels are listed for male and female gerbils that were infected using the IP or SQ infection route. Infection intensity denotes the number of adult worms recovered per successfully infected gerbil. Differences in infection intensities were most apparent between infection routes. c) Box and whisker plot shows that the IP infection route led to a median recovery of 78 worms/gerbil. The SQ infection route led to fewer worms per gerbil (2 worms/gerbil). d) Box and whisker plots show the distribution of the worm recovery for IP-infected female and male gerbils. Males had a slightly higher median recovery (median = 80 worms) compared with the female gerbils (median = 77 worms). However, the difference in mean worm recovery is not statistically significant. e) Box and whisker plots show the distribution of the worm recovery for SQ female and male gerbils. Males had a slightly higher median recovery (median = 2.5 worms) compared with the female gerbils (median = 1.0 worm). Similar to the IP infection results, the difference in mean worm recovery is not statistically significant. f) Box and whisker plots show the microfilaremia levels of SQ-infected male and female gerbils. Microfilaremia levels were higher in SQ-infected male gerbils (median = 5.65 mf/20 µL) compared with the female gerbils (median = 0 mf/20 µL). The difference in microfilaremia levels was found to be statistically significant (*P*-value = 1e−04). The *y*-axis for c, d, e, and f), refers to the total number of parasites recovered per gerbil which are also shown as dots overlayed on the box and whisker plots.

The SQ infection consisted of 150 larvae injected into the gerbil, with 22 male and 32 female gerbils infected. Five SQ-infected gerbils (3 female, 2 male) were removed from the study and analyses for various reasons (Supplementary Table 1). In the remaining 49 SQ-infected gerbils (20 males; 29 females), there was higher prevalence of patent infection in males (18/20; 90%) relative to females (8/29; 28%), as indicated by the presence of microfilariae in a blood draw performed at least 121 d postinfection (*χ*² = 16.09, df = 1, *P* = 6.03e−05) (Fig. 1a).

The presence of microfilariae indicates that adult worms have developed, mated, and reproduced, an important requirement for patent infections. Establishing infection success from a blood draw is both practical and rigorous as it can be measured prior to necropsy. Additionally, locating all adults upon necropsy can be difficult, as observed with gerbils that had microfilariae in the blood, but no adults were identified during necropsy (Supplementary Fig. 1). These gerbils are counted as infected and likely do contain adult worms as indicated by the presence of microfilariae. Gerbils that contain adult worms and no microfilariae are not counted as having a patent infection, since absence of microfilariae indicates that the parasites are not actively reproducing, a requirement for patency. However, male-biased infection is still observed if we define infection as the presence of worms of any life stage, not just microfilariae (*χ*² = 4.2769, df = 1, *P* = 0.03863).

The IP infection method involved injecting 400 larvae into the peritoneal cavity of the gerbil (Mutafchiev et al. 2014). All 7 male and 7 female gerbils were confirmed to be infected. Since microfilariae do not enter the bloodstream with IP infections, successful infection was defined as having adult worms of either sex and/or microfilariae present in the peritoneal cavity at necropsy.

Infection intensity is defined as the number of parasites isolated per successfully infected host. This metric is an important measure to understanding infection success rate and propagation. As expected, infection intensities were significantly higher (*t*-test, *P* = 4.7e−05) in IP-infected gerbils than in SQ-infected gerbils (Fig. 1b and c). The mean infection intensity was similar and not statistically significant between male hosts and female hosts in IP-infected gerbils as well as the SQ model (Fig. 1b, d, and e). Using all SQ-infected gerbils, significantly higher microfilaria (mf) concentrations (mf/20 µL) were found in male gerbils than in female gerbils (Fig. 1b and f). Based on the SQ infection success rate from the 20 male gerbils and 29 female gerbils, a power calculation suggested that 83 gerbils per group would have been required to obtain a statistically significant difference in adult worm recovery (80% power and a statistical cutoff *P*-value < 0.05). A prior study (Wesley 1973) of ∼40 gerbils per group found statistically significant differences, but differed in having a higher adult worm recovery rate per male gerbil (Supplementary File 1). All statistical tests are summarized in Supplementary Table 4.

### Simultaneous analysis of gene expression for host sex, infection mode, and life stage

To test if the gene expression of parasites recovered from male gerbils is the same as those from female gerbils, we undertook a differential expression analysis of adult male worms, adult female worms, and microfilaria from male and female gerbils infected using either SQ infection or IP infection. Our initial goal was to sequence the RNA of litter-matched male and female gerbils where all 3 life stages were recovered. While litter-matched IP-infected males and females were used, the low infection frequency and difficulties in worm recovery precluded this for the SQ-infected gerbils. Only 6 SQ-infected female gerbils and 10 SQ-infected male gerbils had all 3 life stages successfully isolated.

Strand-specific Illumina RNA-Seq libraries were sequenced from the 36 selected samples (Supplementary Table 3) with, on average, 164 million 2 × 100-bp reads per sample. At most, 0.3% of all mapped reads mapped to the *Wolbachia* endosymbiont (Supplementary Fig. 2a). Six SQ microfilariae samples were removed from further consideration as the vast majority of reads belonged to the gerbil (Supplementary Fig. 2b) and a rarefaction analysis indicated that they have insufficient reads (Supplementary Fig. 2c). While the IP microfilariae samples did contain some gerbil reads, more than 50% of the mapped reads mapped to the *B. pahangi* genome (Supplementary Fig. 2b). All IP samples and adult SQ samples had acceptable levels of gerbil reads and reached acceptable levels of saturation in the rarefaction analysis (Supplementary Fig. 2b and c).

**Fig. 2.**
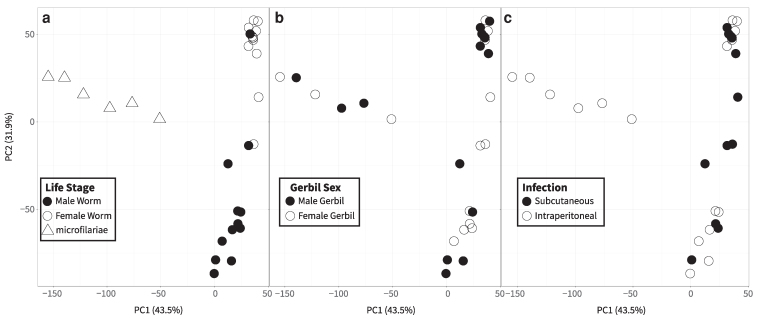
Samples separate by nematode life stage in principal components analysis. Using FactomineR, a principal components analysis was generated using the *z*-score transformation of the log_2_(TPM) values for the 7,384 differentially expressed genes. The first 2 principal components were plotted using ggplot2. The principal components analysis result was then coded to denote a) life stage, b) gerbil sex, or c) infection route. The samples separate, in the first principal and second principal components, by life stage and not by gerbil sex or Infection route.

**Fig. 3.**
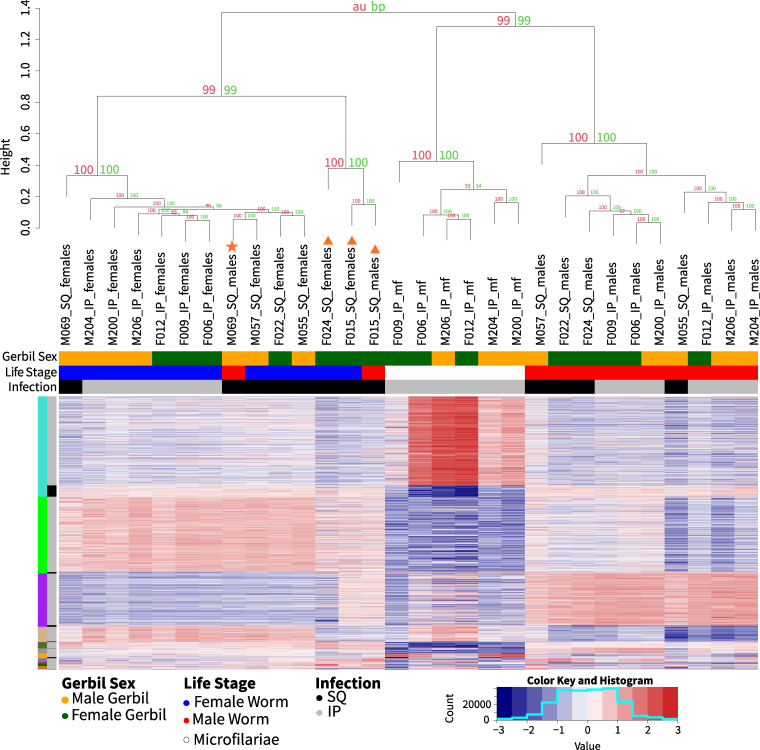
Heatmap and dendrogram of 7,384 differentially expressed genes. Using Weighted Gene Correlation Network Analysis (WGCNA), the 7,384 differentially expressed genes were divided into 16 expression clusters. The genes are shaded based on a *z*-score normalization of the log_2_(TPM) values of the 7,384 differentially expressed genes. The differentially expressed genes were identified using edgeR. The outer column of row colors denotes the WGCNA cluster, while the second column of row colors denotes if those genes match the main pattern (gray) or the inverse pattern (black) for that WGCNA cluster. There are 3 color-coded rows for gerbil sex, nematode life stage, and infection route for each column of data denoting sample classification according to the color key at the bottom of the figure. In the dendrogram generated by pvclust, the left/red values are the approximately unbiased (au) values and right/green values are the bootstrap probabilities. The orange triangles denote the 2 female SQ samples and 1 male SQ sample that formed a separate cluster from their respective life stages. The orange star denotes the male sample that has a very similar transcriptome to the females and clusters with them.

There were 7,384 differentially expressed genes identified with a generalized log-linear model in EdgeR v3.30.4 (Robinson et al. 2010) when classifying samples on 3 variables–gerbil sex (male/female), infection model (IP/SQ), and worm life stage (microfilariae/adult male/adult female). After normalizing the counts of only differentially expressed genes to a *z*-score of log_2_-transformed transcripts-per-million (TPM), a principal components analysis (PCA) and dendrogram of the remaining thirty samples were generated with factomineR v2.6 (Lê et al. 2008) and pvclust v2.2-0 (Suzuki et al. 2019), respectively. In the PCA, the first 2 principal components accounted for ∼75% of the variation and the samples clustered strongly by life stage (Fig. 2a), as previously described in the related *B. malayi* (eg Holt and Dunning Hotopp 2024). Within the first principal component, the samples separate by adults and microfilariae. In the second principal component, the adult female worms and microfilariae overlap. This is consistent with previous results in *B. malayi* where there is overlap in females and microfilariae as mature females are full of developing embryos and microfilariae (Choi et al. 2011; Chung et al. 2019). When looking at the other 2 variables (gerbil sex and infection route), the samples did not form any further clusters (Fig. 2b and c). Likewise, in the dendrogram, there are 3 well-supported clusters (Fig. 3, Supplementary Fig. 3) roughly corresponding to microfilariae, adult male worms, and adult female worms. The dendrogram shows no separation based on infection route or gerbil sex. The exceptions to these clusters are 1 male and 2 female SQ infection samples that form a separate cluster (F024_SQ_females, F015_SQ_females, F015_SQ_males) (Fig. 3, each denoted with a triangle) and a single SQ male sample that clusters with the females with strong support (M069_SQ_males) (Fig. 3, denoted with a star). The 7,384 differentially expressed genes were sorted into 16 clusters using WGCNA, which separated the samples largely based on nematode life stage (Supplementary File 2, Fig. 3).

A linear mixed model allows for a quantitative summary of the drivers of transcriptomic variation within a differential expression analysis. This model was used to provide quantification of the amount of transcriptomic variance attributed to the 3 variables in this study: gerbil sex (male/female), infection model (IP/SQ), and worm life stage (microfilariae/adult male/adult female). Gerbil litter was also included for the IP gerbils. Any variation that cannot be explained by 1 of the 4 variables is classified as residuals. Genes in a given dataset are fit to a regression model and the relative contribution of each variable to the gene expression variation is calculated. The linear mixed model was implemented using the R packages variancePartition v1.26.0 (Hoffman and Schadt 2016) and limma v3.52.4 (Ritchie et al. 2015). This analysis was run using counts generated by HTSeq-Counts for all genes, not just those determined to be differentially expressed.

**Fig. 4.**
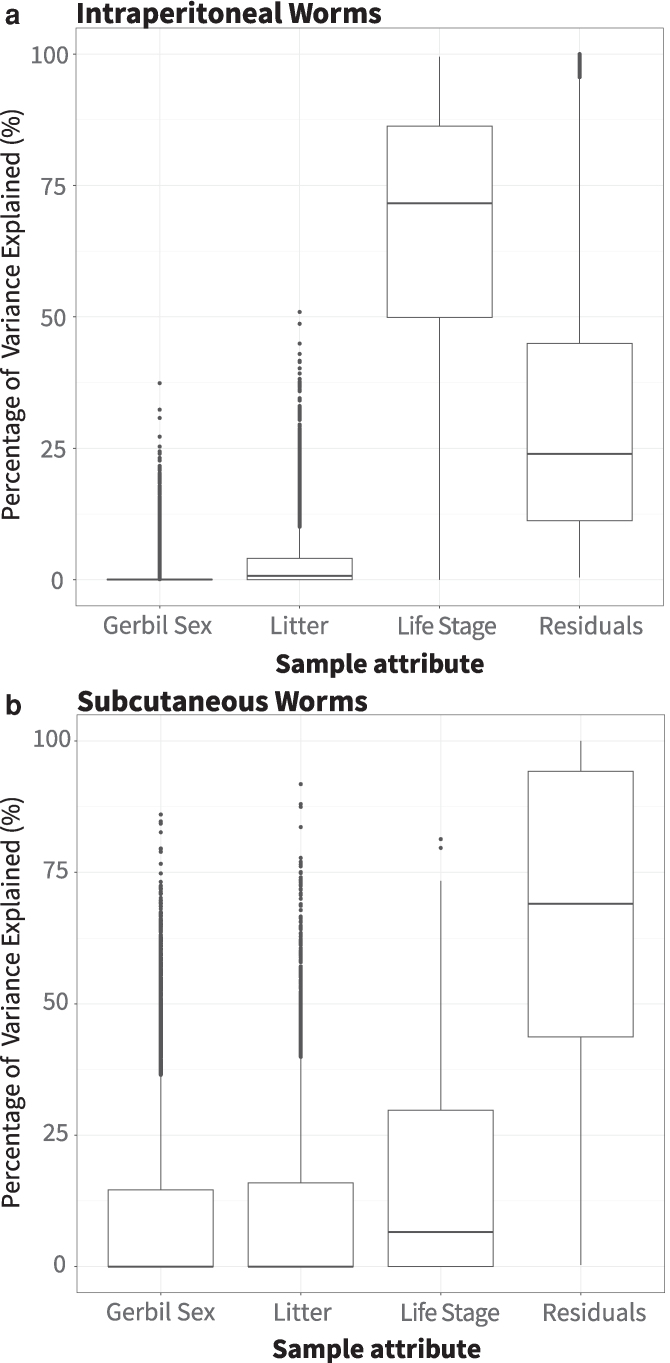
Worms from SQ-infected gerbils exhibit greater transcriptional variation. A linear mixed model, using the counts for all genes, highlights the variation in gene expression that can be attributed to 3 variables for the a) IP samples and the b) SQ samples. Life stage is the primary driver of gene variation in IP worms, while SQ worms show little separation based on life stage. SQ worms show too much variation for reliable differential expression in this study. The plots were generated with ggplot2.

The linear mixed model analysis of worms in IP-infected gerbils shows that life stage is the variable that is driving the transcriptional differences between these worms. This is expected and has been demonstrated previously in the related *B. malayi* (eg (Holt and Dunning Hotopp 2024)). However, this was not the case for worms from SQ-infected gerbils. For the IP worms, life stage accounted for 71.6% of sample variation, followed by residuals at 23.9%, then by litter (0.73%) and host sex (<<0.0001%) (Fig. 4a). For the SQ worms, life stage accounts for 6.6% of sample variation and residuals accounted for 69% of sample variation (Fig. 4b). Residuals denote an unaccounted-for factor (or potentially multiple unaccounted for factors) that is separating samples, indicating that there is a large amount of intrasample variation (eg the SQ male worms do not show consistent expression patterns) (Fig. 4b). Rerunning the differential expression analysis using only SQ worms produced 0 differentially expressed genes.

### Pairwise analyses of single variables of gene expression for host sex, infection mode, and life stage

**Fig. 5.**
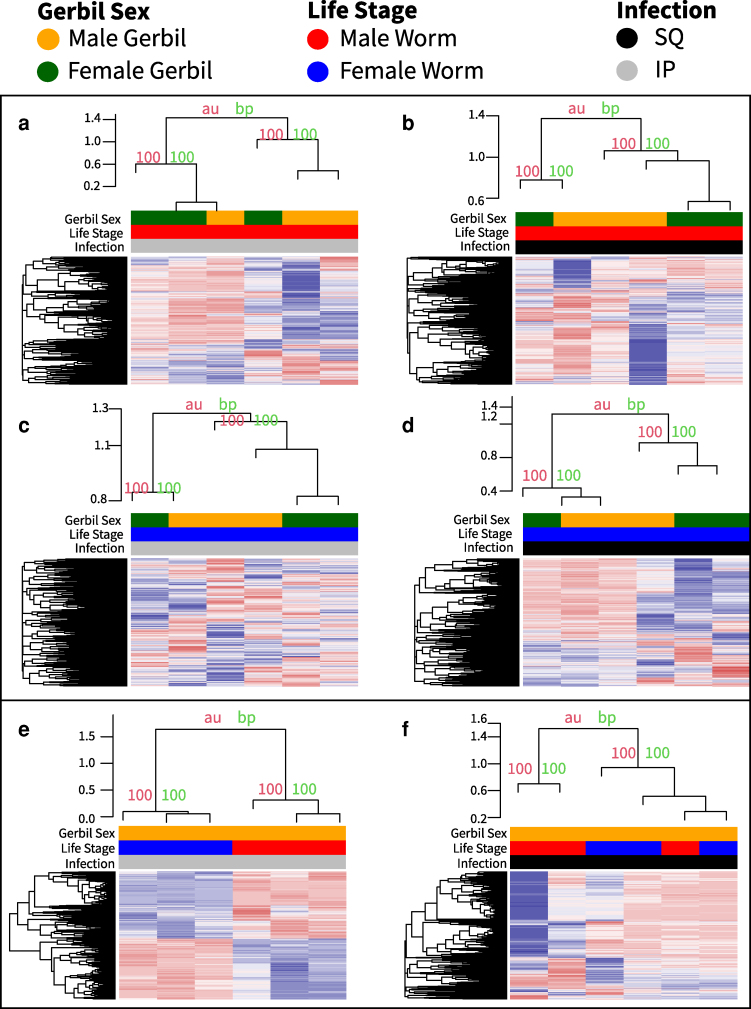
Lack of differential expression between male and female gerbils and a lack of differential expression in worms from SQ-infected gerbils. To assess specific variables in isolation, pairwise comparisons were examined for: a) male worms from IP-infected male and female gerbils; b) male worms from SQ-infected male and female gerbils; c) female worms from IP-infected male and female gerbils; d) female worms from SQ-infected male and female gerbils; e) male and female worms from IP-infected male gerbils; f) male and female worms from SQ-infected male gerbils. The analysis in panel e) uses the *z*-score normalization of the log_2_(TPM) values for the 5,462 differentially expressed genes. But since there are no differntially expressed genes in the other comparisons, for the other panels, the *z*-score normalizations of the log_2_(TPM) values are used for all of the genes that passed the edgeR CPM filter. The differentially expressed genes were identified using edgeR. There are 3 color-coded rows for gerbil sex, nematode life stage, and infection route for each column of data denoting sample classification according to the key at the top of the figure. In the dendrogram, approximately unbiased (red/left) values, and bootstrap support (green/right) values were generated with pvclust and the heatmaps were drawn with heatmap.3.

To rule out that differential expression was obscured in the multivariate analysis, we repeated the analysis using pairwise analyses focusing on single variables at a time (ie comparing male worms from IP-infected male gerbils to male worms from IP-infected female gerbils). We still found no differentially expressed genes associated with host sex or infection route (Fig. 5a to d), whether we adjusted for multiple testing or not. The reason for the lack of differentially expressed genes is different for the IP and SQ infections. Whether from male or from female gerbils, worms from IP infections have the same strongly reproducible transcriptional response, which is similar to a transcriptional response that has been observed across many studies for the related *B. malayi* (Choi et al. 2011; Grote et al. 2017; Chung et al. 2019; Holt and Dunning Hotopp 2024). However, this is not the case for worms from the SQ-infected gerbils. The worms from SQ-infected gerbils show a tremendous amount of variation compared with worms from IP infections, possibly due to different microenvironments or due to the increased handling required for extracting them from the lymphatics.

Consistent with the homogeneity of the response in the IP model, 5,462 genes were found to be differentially expressed between male and female worms in male gerbils (Fig. 5e, Supplementary Fig. 4a) while 7,055 genes were differentially expressed between male and female worms from female gerbils (Supplementary Fig. 4b). Between these 2 sets of differentially expressed genes, there are 5,295 differentially expressed genes that overlap. However, due to the extensive variation in the transcriptomes of worms from SQ-infected gerbils, no differentially expressed genes were identified in comparisons of male and female worms (Fig. 5f, Supplementary Fig. 4c, d). It is the substantial variation between SQ worms that hinders our ability to accurately identify differentially expressed genes under these conditions for these sample sizes. This is consistent with the analysis with variancePartition described above, where most of the variance is in the residuals (Fig. 4). Therefore, we conclude that gerbil sex has no effect on transcription in the IP infection model but cannot reach a conclusion for the SQ infection model. The tremendous transcriptional variation in the SQ model is in stark contrast to the homogeneity found in the IP model.

### Reduction of SQ sample variation

In contrast to the pooled IP samples, relatively few worms were recovered from the SQ-infected gerbils, with 5 out of 12 SQ samples consisting of only 1 worm (Supplementary Fig. 5). To assess the impact this may have had, the SQ samples were merged (based on life stage and host sex) and then downsampled to a similar number of reads as a single IP sample. These combined SQ samples were included with the IP samples in a differential expression analysis. This analysis revealed a reduction in the intrasample variation that was displayed in the original analysis (Supplementary Fig. 5). Fewer differentially expressed genes were identified (5,857) compared with the previous analysis (7,384). However, as this reanalysis only included adult male and adult female samples and did not include microfilariae, fewer differentially expressed genes are to be expected. There were 77 significantly enriched functional terms that were identified, and there is significant overlap in these terms compared with the previous analysis. This suggests that in the aggregate the SQ samples are similar to the related IP samples.

SQ infection of animal hosts with *B. malayi* or *B. pahangi* leads to worms being located in various lymphatic tissues in the body, such as the heart/lungs, genital lymphatics, and SQ layers of the skin (Ash and Riley 1970a, 1970b; Ah and Thompson 1973; Athisaya Mary et al. 2006). We could not identify a correlation between the body site where the SQ worms were extracted from and the worm's transcriptome. Although most of the samples were isolated from the heart/lung region of the host, the transcriptional variation of the SQ samples prevented any comparisons between worms isolated from the same or different body sites.

### No host sex effect on the *B. pahangi* transcriptome

Most studies on *B. malayi* use nematodes recovered from IP-infected male gerbils, and this is the only material available from the NIAID-funded FR3 resource center (Michalski et al. 2011). This study suggests that worms of the same life stage recovered from IP-infected male and female gerbils were transcriptionally indistinguishable, supporting the use of solely male gerbils in research. The IP infection route is frequently preferred as it provides a large number of adult nematodes per gerbil. Additionally, the worms isolated from the infected gerbils are robust with a reliable, consistent, and homogenous transcriptional response (Holt and Dunning Hotopp 2024).

### Significantly enriched functional terms between *B. pahangi* and *B. malayi*

The functional terms associated with the *B. pahangi* clusters are similar to functional terms previously described in *B. malayi* (Supplementary File 3, Supplementary File 4) (Choi et al. 2011; Grote et al. 2017; Chung et al. 2019), including (i) in microfilariae: GPCR activity, proteolysis, and various voltage-gated potassium channel terms; (ii) in adult females: DNA-binding transcription factor activity and sequence-specific DNA binding; and (iii) in males: PapD-like superfamily term, major sperm protein domain, tyrosine-specific protein phosphatase, and protein kinase activity (Supplementary File 3). There are significantly enriched functional terms that differ between the 2 species, including enrichment in *B. pahangi* of (i) various neurotransmitter-gated ion-channel terms, potassium ion transport, and CUB domain in microfilariae, (ii) GTP binding, protein binding, and GTPase activity in adult females, and (iii) dephosphorylation in adult males and enrichment in *B. malayi* of (i) DNA binding, zinc finger C2H2-type, and aminoacyl-tRNA ligase activity in microfilariae; (ii) EGF-like domain and zinc finger in adult females; and (iii) protein-kinase-like domain super family, protein-tyrosine phosphatase, and metal-dependent phosphatases in adult males and (Supplementary Files 3, 4, Supplementary Fig. 6).

### *B. pahangi* genome annotation

To perform this differential gene expression analysis, genome annotation of the 96 Mb *B. pahangi* FR3 genome (JAAVKF00000000) (Mattick et al. 2020) was necessary. There is 80 Mb of *B. pahangi* FR3 genome sequence that is assigned to the nearly complete autosomes and chromosome X, similar to the 82 Mb for *B. malayi* (Foster et al. 2020; Tracey et al. 2020). The remaining 16 Mb in *B. pahangi* and 6 Mb in *B. malayi* are largely repetitive sequences corresponding to gaps that are mostly in the Y chromosome, which is unresolved in both organisms.

In this study, we predicted gene structures for *B. pahangi* with BRAKER using a combination of short read Illumina RNA-Seq and long read ONT direct RNA sequencing (Supplementary Table 2) (Stanke and Morgenstern 2005; Stanke et al. 2006, 2008; Li et al. 2009; Barnett et al. 2011; Lomsadze et al. 2014; Buchfink et al. 2015; Hoff et al. 2016, 2019; Mattick et al. 2020; Bruna et al. 2021). Although there is reduced sequencing depth, the long ONT direct RNA reads are better at resolving gene structures and isoforms compared with short Illumina reads. A second structural annotation was predicted using the conserved protein domains from OrthoDB with a final structural annotation generated with a merge of the 2 initial annotations using TSEBRA (Altschul et al. 1990; Lomsadze et al. 2005; Stanke and Morgenstern 2005; Stanke et al. 2006, 2008; Gotoh 2008, 2014; Camacho et al. 2009; Iwata and Gotoh 2012; Buchfink et al. 2015; Hoff et al. 2016, 2019; Kriventseva et al. 2019; Bruna et al. 2020, 2021; Mattick et al. 2020). The functional annotation, which describes the predicted function of RNAs and proteins encoded in those transcripts, was generated using evidence from HMMER/Hmmscan, RNAmmer v1.2, tRNAscan-SE v2.0.3, TMHMM v2.0 and Rapsearch2. This evidence was evaluated and applied to gene models using BioCode v0.10.0 and Attributor, resulting in a functionally annotated GFF3-formatted file (Lowe and Eddy 1997; Krogh et al. 2001; Lagesen et al. 2007; Josh Orvis; J Orvis; Zhao et al. 2012). Interproscan v5.56-89.0 was used to assign IPR and GO terms to each gene model (Jones et al. 2014).

**Fig. 6.**
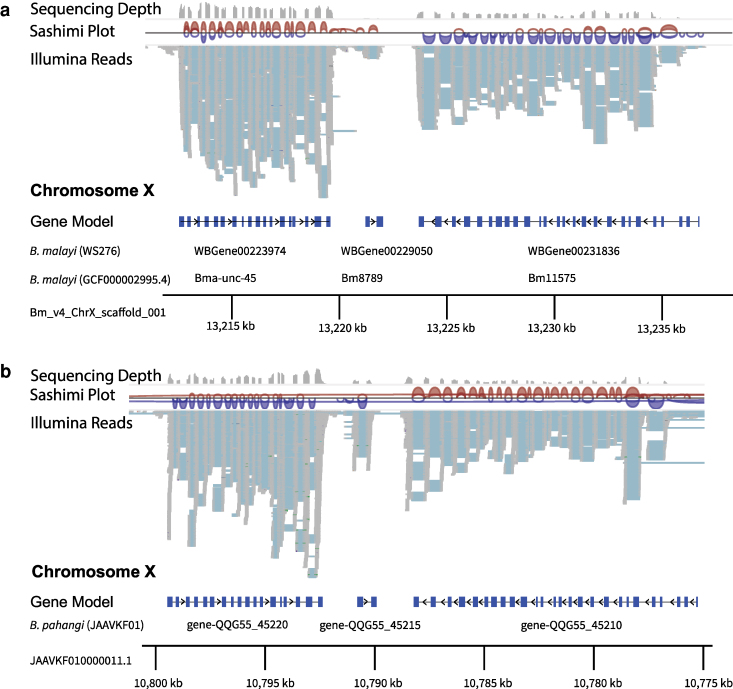
Orthologous genes between *B. malayi* and *B. pahangi.* Annotated Integrative Genomics Viewer (IGV) image from orthologous regions between a) *B. malayi* and b) *B. pahangi* along with Illumina reads and gene predictions. The panels are oriented so that the orthologous genes are vertically aligned. WBGene00223974 is orthologous with gene-QQG55_45220, WBGene00229050 is orthologous with gene-QQG55_45215, and WBGene00231836 is orthologous with gene-QQG55_45210. The sashimi plots show the proper splicing between the reads.

On the contigs attributed to autosomes or chromosome X, 10,316 *B. pahangi* genes were annotated with 2,878 (28%) genes where all the transcripts are annotated as a hypothetical protein. For *B. malayi*, there are 10,373 genes with 3,567 (34%) annotated as hypothetical proteins. The *B. malayi* and *B. pahangi* annotations are similar in having genes with on average ∼12 CDS/gene, ∼150 bp CDS, and ∼2,500 multi-transcript genes annotated (Table 1). Visual inspection of the annotation for *B. pahangi* and *B. malayi* in regions with synteny reveals similar gene predictions with similar intron/exon structure (Fig. 6).

**Table 1.** Annotation differences between *B. pahangi* and *B. malayi*.

Annotation feature statistics	*B. pahangi*	*B. malayi*
NCBI WGS accession	JAAVKF01	CAAKNF01
Assembly accession	GCA_012070555.1	GCF_000002995.4
Number of genes with multiple transcripts	2,546	2,589
Number of genes with multiple transcripts on autosomes and x chromosome only	1,972	2,452
Number of total gene features	11,310	10,905
Number of total gene features on autosomes and X chromosome only	10,316	10,373
Number of CDS features per gene	12.5	12.8
Number of inferred intron features per gene	11.2	11.5
Average CDS feature length (bp)	147	148

The untranslated regions (UTRs) are largely unannotated in both genomes. While direct RNA-sequencing data should enable the identification and annotation of the UTRs, most UTRs overlap with a noncoding RNA that are often on the same strand, which confounded the accurate prediction of the UTRs. Therefore, the differential expression analysis was undertaken with the generated and deposited protein-centric annotation (accession JAAVKF01). This is similar to the numerous differential gene expression analyses undertaken on *B. malayi* (Holt and Dunning Hotopp 2024), which is also protein-centric likely for an analogous reason.

## Discussion

Studies on host-sex effects have historically focused on infection rates, sociological practices, immune responses, worm recovery rates, or the host transcriptome. A review of sex and parasitic diseases (specifically leishmaniasis, Chagas disease, amebiasis, malaria, and schistosomiasis) focused on clinical infection bias and host immune response differences between male and female hosts in response to infection (Honecker et al. 2026). Less is known about how host-sex effects could influence the parasite's transcriptome.

Worms recovered from IP infections in male gerbils had similar transcriptional profiles as their counterparts from female gerbils. As such, there does not appear to be an effect of host sex on gene expression for adult male and adult female worms recovered from the IP infection model. Worms recovered from SQ infections were transcriptionally variable, precluding the identification of differentially expressed genes between adult males and adult females or between worms recovered from male and female gerbils. The cause of SQ sample variation may be from differential exposure to the host immune system, different tissue environments throughout their lifecycle, lower amounts of RNA input, and/or prolonged mechanical forces during necropsy, as worms from SQ infection are more difficult to recover. The transcriptional variability between SQ worms limited the interrogation of the transcriptional response to host sex for adult worms. Further investigation of the transcriptional variability of worms at different body sites could clarify this, with spatial transcriptomics having great potential in this regard.

The transcriptional variation in SQ samples is unlikely to be due to the maturity of the female parasites. The prepatent period, defined as the number of days before microfilariae were present in the blood, was used to determine the maturity of the infection and of the female parasites. The mean prepatent period in SQ-infected gerbils is 57 to 84 d with *B. pahangi* (Ash and Riley 1970a). In the study presented here, the earliest necropsy was performed at 123 d postinfection and the latest necropsy performed 309 d postinfection (median 226 d). Samples did not cluster based on the age of gerbil at the time of infection or the length of infection.

Overall, we established that in the IP model, there are no discernible differences in the transcriptomes of worms of *B. pahangi* in 3 key life stages from male or female gerbils. This lack of impact of host sex on the transcriptome of worms isolated from IP-infected gerbils reinforces the use of male gerbils as the primary rearing host for these parasites.

## Supplementary Material

jkag073_Supplementary_Data

## Data Availability

The data generated and used in this study has been deposited in the Sequence Read Archive repository. The RNA-Seq reads are available under the BioProject accession PRJNA817251. The differential expression and counts matrices are available under the Gene Expression Omnibus accession GSE312060. The sample SRX accession codes used in the genome annotation are available in Supplementary Table 2 and the SRX accession codes used in the differential expression analysis are available in Supplementary Table 3. Source code and supplemental data files to reproduce this analysis are available at https://github.com/christopher-holt/bpahangi_host_sex_effect (https://zenodo.org/doi/10.5281/zenodo.19356197). The genome annotation is available under the updated GenBank accession JAAVKF010000000. The full differential expression result tables are available in Supplementary Table 5. Supplemental material available at G3 online.
