# Supplementary material for: Gene regulation in response to host sex and infection route in Brugia pahangi with new genome annotation
Source: G3 (Bethesda). 2026 Apr 15;16(6):jkag073. doi: 10.1093/g3journal/jkag073 (PMC13261522; doi:10.1093/g3journal/jkag073)
Supplement: jkag073_Supplementary_Data [file jkag073_Supplementary_Data.zip › Supplemental_Material_Legends_G3-2026-406658.docx]

## Supplementary Files

### Supplementary Figure 1. Presence of Worm Life Stage in SQ-Infected Gerbils

A graphical summary of worm recovery in SQ-infected gerbils shows observed adult worms or microfilariae marked by a shaded red rectangle, while no observation of those life stages are unshaded. There were 16 gerbils where all three life stages were recovered. When female worms were present, there were always males and/or microfilariae recovered. There were 11 gerbils where only microfilariae or male worms were recovered. The values included refer to the number of adult worms recovered per gerbil and the number of microfilariae per 20μL of blood.

### Supplementary Figure 2: Rarefaction Curves of *B. pahangi* Samples

**a)** Bar plot showing the percentage of the reads that mapped to the obligate *Wolbachia* endosymbiont of *B. pahangi*. The legend above the sample names denotes the gerbil sex, life stage, or infection route used. The bottom row denotes the infection route: IP (gray) or SQ (black). The middle row denotes the life stage of the sample: adult male (red), adult female (blue), or microfilariae (white). The top row denotes the sex of the gerbil: male (orange) or female (green). **b)** Stacked bar plot showing the percentage of reads that mapped to the gerbil genome (salmon), *B. pahangi* mitochondrial genome (green), *B. pahangi* nuclear genome (light blue), or the *B. pahangi* obligate *Wolbachia* endosymbiont (purple). The legend above the sample names denotes the gerbil sex, life stage, or infection route used. The bottom row denotes the infection route: IP (gray) or SQ (black). The middle row denotes the life stage of the sample: adult male (red), adult female (blue), or microfilariae (white). The top row denotes the sex of the gerbil: male (orange) or female (green). **c)** Rarefaction curves of the 36 *B. pahangi* samples were generated with vegan and rendered with ggplot2. Each curve in the rarefaction is labelled by the sample’s infection route and life stage attributes. Based on this analysis, the microfilarial samples isolated from SQ-infected gerbils had to be removed from the differential expression analysis as they did not reach saturation.

### Supplementary Figure 3: Log Transformed Heatmap and Dendrogram

The 7,384 differentially expressed genes are shaded based on the log_2_(TPM) values. The differentially expressed genes were identified using edgeR. Three rows in the column legend denote sample classification. The bottom row denotes the Infection route: IP (gray) or SQ (black). The middle row denotes the life stage of the sample: adult male (red), adult female (blue) or microfilariae (white). The top row denotes the sex of the gerbil: male (orange) or female (green). The heatmap was generated with [heatmap.3](https://raw.githubusercontent.com/obigriffith/biostar-tutorials/master/Heatmaps/heatmap.3.R) and the dendrogram was generated with pvclust. In the dendrogram generated by pvclust, the red values are the approximately unbiased (au) values and green values are the bootstrap probabilities.

### Supplementary Figure 4: Pairwise Comparisons of *B. pahangi* Samples Comparing Life Stage

Pairwise comparisons were used to compare worms from different life stages, while keeping gerbil sex and infection mode consistent, including: **a)** male and female worms from IP-infected male gerbils where 5,462 differentially expressed genes were detected; **b)** male and female worms from IP-infected female gerbils where 7,055 differentially expressed genes were detected; **c)** male and female worms from SQ-infected male gerbils; and **d)** male and female worms from SQ-infected female gerbils. Differentially expressed genes were identified using edgeR for panels **a** and **b** while panels **c** and **d** contain genes that passed the edgeR CPM filter as no differentially expressed genes were detected. The genes are shaded based on a z-score normalization of the log_2_(TPM) values for the genes in each heatmap. The bottom row in the column legend denotes the infection route: IP (gray) or SQ (black). The middle row denotes the life stage of the sample: adult male (red), or adult female (blue). The top row denotes the sex of the gerbil: male (orange) or female (green). The heatmap was generated with [heatmap.3](https://raw.githubusercontent.com/obigriffith/biostar-tutorials/master/Heatmaps/heatmap.3.R) and the dendrogram was generated with pvclust. In the dendrogram generated by pvclust, the red values are the approximately unbiased (au) values and green values are the bootstrap probabilities.

### Supplementary Figure 5: Combining SQ Samples Reveals a Reduction in Sample Variation

The SQ adult worm samples were merged (based on host sex and life stage) using samtools then downsampled to 100 million reads using picard. The left heatmap, directly adapted from **Figure 3**, shows the heatmap and sample clustering of the 30 sequencing samples. The numbers after the SQ sample name denotes the number of worms used in that sample. The right heatmap contains on the adult male and female samples, with the SQ samples merged and downsampled. The SQ samples have been labelled with either a circle (male worms from SQ-infected female gerbils), a star (male worms from SQ-infected male gerbils), a triangle (female worms from SQ-infected female gerbils), or a square (female worms from SQ-infected male gerbils). A grey shape denotes an SQ sample that clustered where expected, an orange shape denotes an SQ sample clustering differently. The numbers after each of the sample names in the left heatmap indicates the number of worms that were isolated for that sample. The 5,857 differentially expressed genes in the right heatmap are shaded based on the z-score normalization of the log_2_(TPM) values. The differentially expressed genes were identified using edgeR. Three rows in the column legend denote sample classification. The bottom row denotes the Infection route: IP (gray) or SQ (black). The middle row denotes the life stage of the sample: adult male (red), or adult female (blue). The top row denotes the sex of the gerbil: male (orange) or female (green). The heatmap was generated with [heatmap.3](https://raw.githubusercontent.com/obigriffith/biostar-tutorials/master/Heatmaps/heatmap.3.R) and the dendrogram was generated with pvclust. In the dendrogram generated by pvclust, the red values are the approximately unbiased (au) values and green values are the bootstrap probabilities.

### Supplementary Figure 6: Life stage specific expression in *B. malayi* adult males, adult females, and mature microfilariae

There are 9,463 differentially expressed genes identified between *B. malayi* adult males, adult females, and mature microfilariae. They were divided into three clusters by WGCNA and are shaded by the z-score normalization of the log_2_(TPM) values. The shaded legend at the top denotes the life stage: adult female (medium purple), adult male (dark orchid), and mature microfilariae (dark orange). The heatmap was generated with [heatmap.3](https://raw.githubusercontent.com/obigriffith/biostar-tutorials/master/Heatmaps/heatmap.3.R) and the dendrogram was generated with pvclust. In the dendrogram generated by pvclust, the red values are the approximately unbiased (au) values and green values are the bootstrap probabilities.

### Supplementary File 1: Tabular Summary of Worm Recoveries from a Previous Study Published in a Student Thesis (Wesley, 1973)

### Supplementary File 2: WGCNA Composition of 7,384 Differentially Expressed Genes

### Supplementary File 3: Significantly Enriched Functional Terms in *B. pahangi*

### Supplementary File 4: Significantly Enriched Functional Terms in *B. malayi*

### Supplementary Table 1: Gerbils Removed from The Study

### Supplementary Table 2: Samples used for Genome Annotation

### Supplementary Table 3: Samples used for Differential Expression

### Supplementary Table 4: Summary of Statistical Tests

### Supplementary Table 5: Differential Expression Tables
