# Supplementary material for: Gene regulation in response to host sex and infection route in Brugia pahangi with new genome annotation
Source: G3 (Bethesda). 2026 Apr 15;16(6):jkag073. doi: 10.1093/g3journal/jkag073 (PMC13261522; doi:10.1093/g3journal/jkag073)
Supplement: jkag073_Supplementary_Data [file jkag073_Supplementary_Data.zip › Supplementary_Figure_1_G3-2026-406658.pdf]

Worms Observed | No Worms Observed

Female Worm	Male Worm	Microfilariae	
1	1	0.5	F022_SQ
3	2	0.3	F016_SQ
2	1	0.5	M070_SQ
1	2	2.5	F024_SQ
1	3	1.5	F044_SQ
1	2	12	M051_SQ
1	3	7	M069_SQ
2	1	5.3	F020_SQ
2	1	5.3	M062_SQ
2	1	10.5	M071_SQ
3	1	10	M056_SQ
3	2	1.3	F015_SQ
3	4	14	M057_SQ
3	2	17	M050_SQ
2	2	18	M055_SQ
4	2	6	M067_SQ
1	0	2.5	M052_SQ
1	0	1	F017_SQ
1	0	5.3	M066_SQ
2	0	3.3	M068_SQ
2	0	11	M065_SQ
1	2	0	F043_SQ
1	1	0	F013_SQ
1	7	0	F037_SQ
0	1	0	F036_SQ
0	1	0	F030_SQ
0	2	0	F029_SQ
0	2	0	F040_SQ
0	2	0	F042_SQ
0	5	0	F032_SQ
0	0	1	M064_SQ
0	0	0.3	F019_SQ
0	0	1	M063_SQ
0	0	2	M061_SQ
0	0	6	M060_SQ
0	0	0	F021_SQ
0	0	0	F018_SQ
0	0	0	F025_SQ
0	0	0	F026_SQ
0	0	0	F028_SQ
0	0	0	F031_SQ
0	0	0	F033_SQ
0	0	0	F034_SQ
0	0	0	F035_SQ
0	0	0	F038_SQ
0	0	0	F039_SQ
0	0	0	F041_SQ
0	0	0	M053_SQ
0	0	0	M054_SQ
