# Supplementary material for: Gene regulation in response to host sex and infection route in Brugia pahangi with new genome annotation
Source: G3 (Bethesda). 2026 Apr 15;16(6):jkag073. doi: 10.1093/g3journal/jkag073 (PMC13261522; doi:10.1093/g3journal/jkag073)
Supplement: jkag073_Supplementary_Data [file jkag073_Supplementary_Data.zip › Supplementary_Figure_3_G3-2026-406658.pdf]

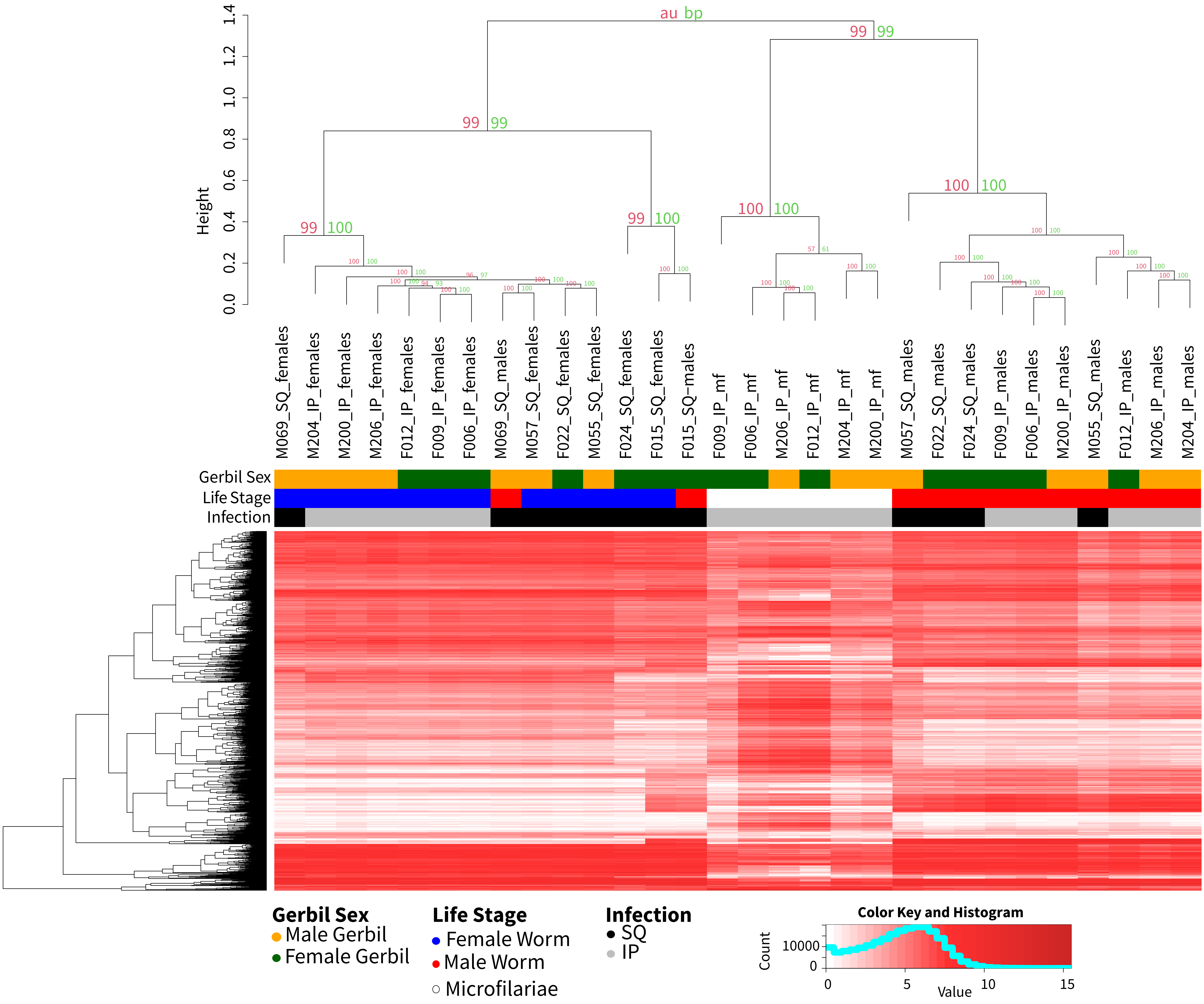

au bp
Height
0.0
0.2
0.4
0.6
0.8
1.0
1.2
1.4
M069_SQ_females
M204_IP_females
M200_IP_females
M206_IP_females
F012_IP_females
F009_IP_females
F006_IP_females
M069_SQ_males
M057_SQ_females
F022_SQ_females
M055_SQ_females
F024_SQ_females
F015_SQ_females
F015_SQ–males
F009_IP_mf
F006_IP_mf
M206_IP_mf
F012_IP_mf
M204_IP_mf
M200_IP_mf
M057_SQ_males
F022_SQ_males
F024_SQ_males
F009_IP_males
F006_IP_males
M200_IP_males
M055_SQ_males
F012_IP_males
M206_IP_males
M204_IP_males
Gerbil Sex
Life Stage
Infection
Gerbil Sex
Male Gerbil
Female Gerbil
Life Stage
Female Worm
Male Worm
Microfilariae
Infection
SQ
IP
Color Key and Histogram
Count
10000
0
Value
0
5
10
15
