# Supplementary figures and images for: Gene regulation in response to host sex and infection route in Brugia pahangi with new genome annotation
Source: G3 (Bethesda). 2026 Apr 15;16(6):jkag073. doi: 10.1093/g3journal/jkag073 (PMC13261522; doi:10.1093/g3journal/jkag073)

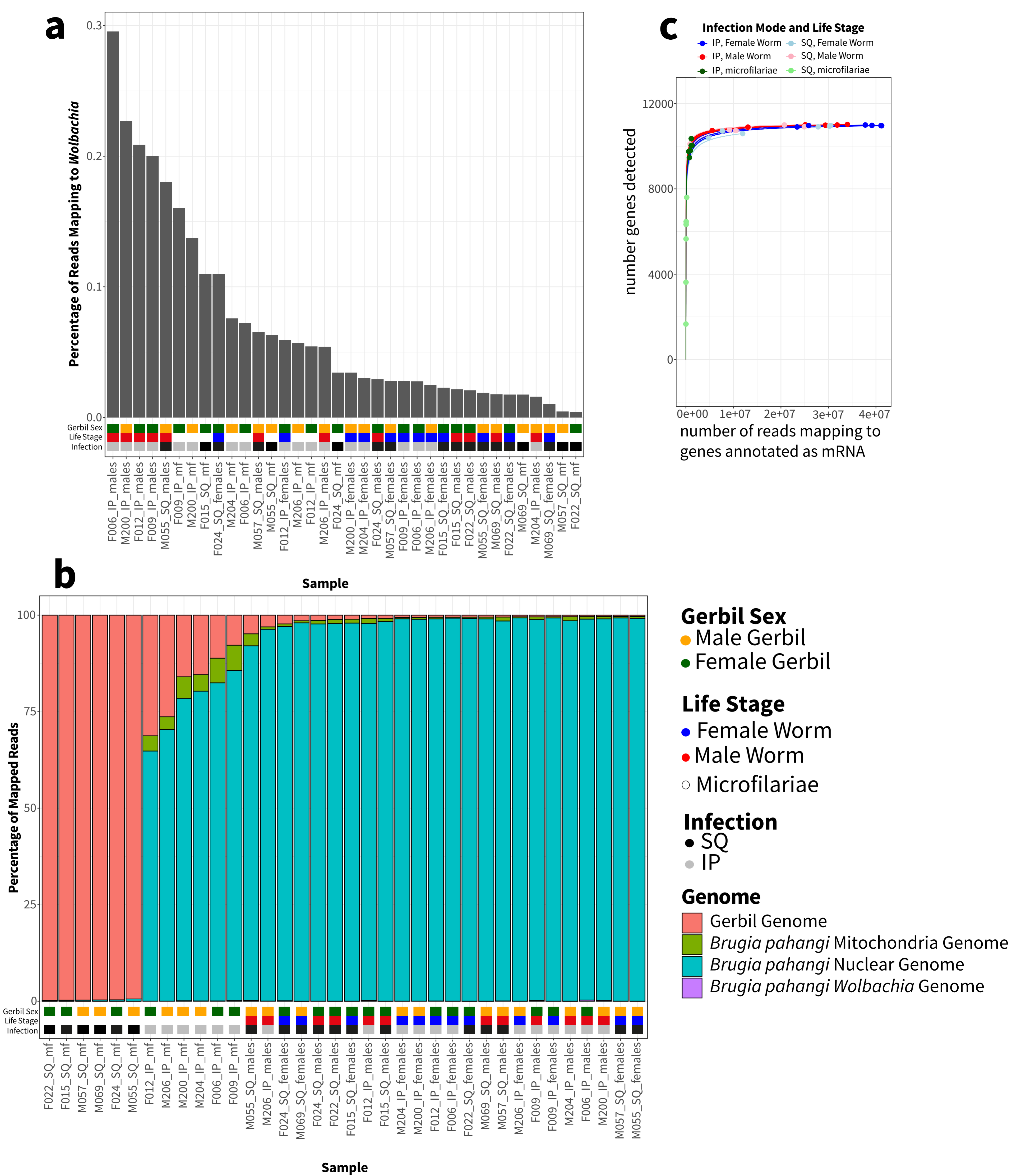

Supplement: jkag073_Supplementary_Data [file jkag073_Supplementary_Data.zip › Supplementary_Figure_2_G3-2026-406658.pdf]

## Gerbil Sex

● Male Gerbil

● Female Gerbil

## Life Stage

● Female Worm

● Male Worm

## Infection

● SQ

● IP

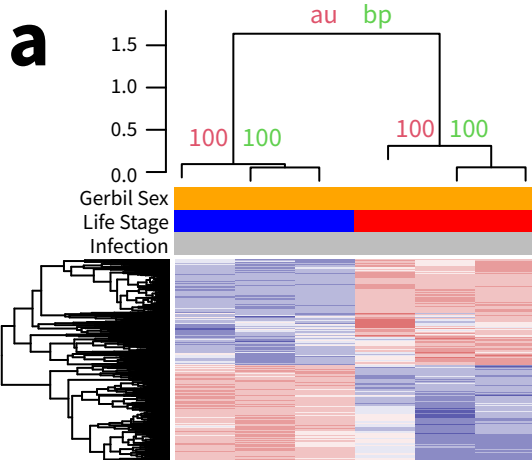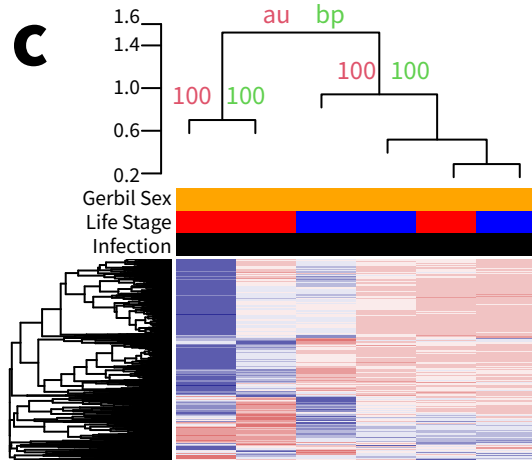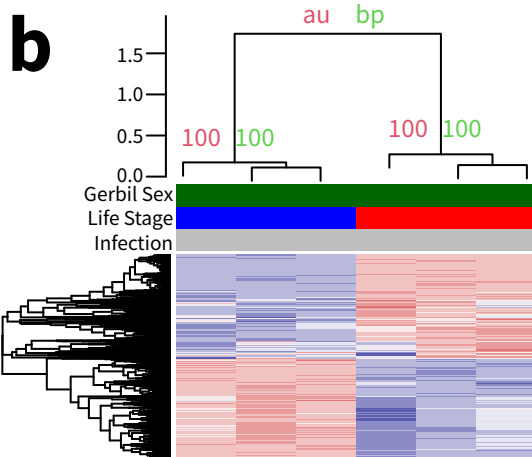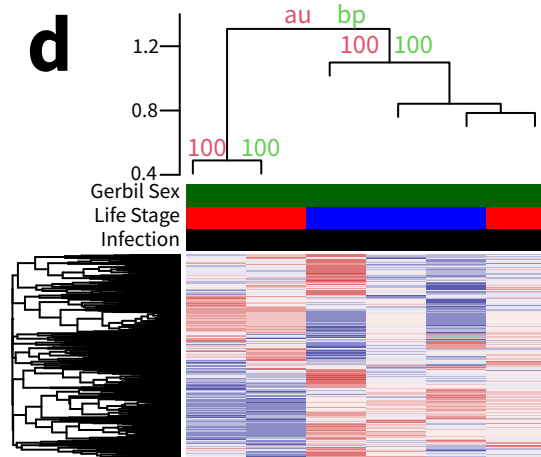

Supplement: jkag073_Supplementary_Data [file jkag073_Supplementary_Data.zip › Supplementary_Figure_4_G3-2026-406658.pdf]

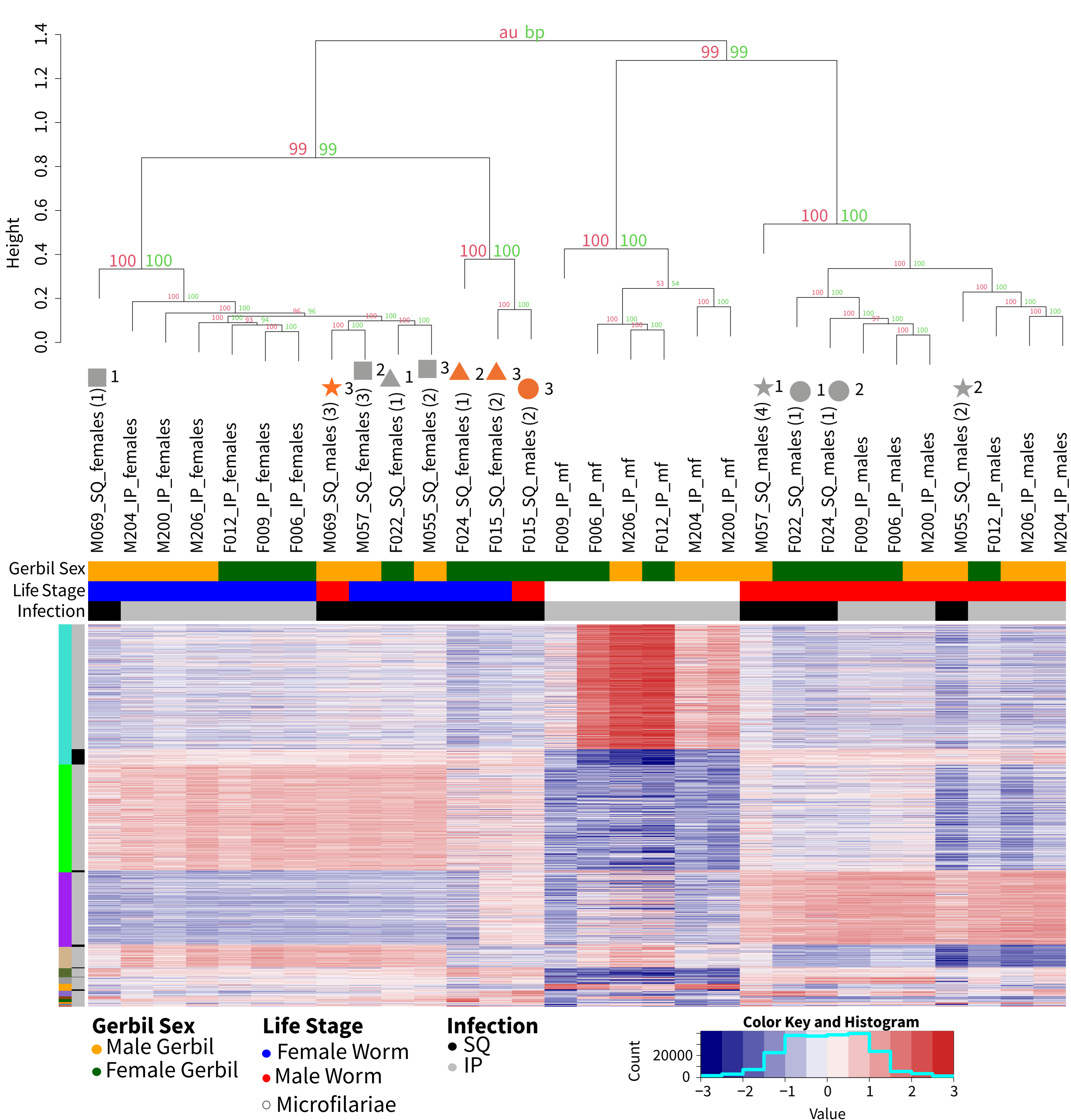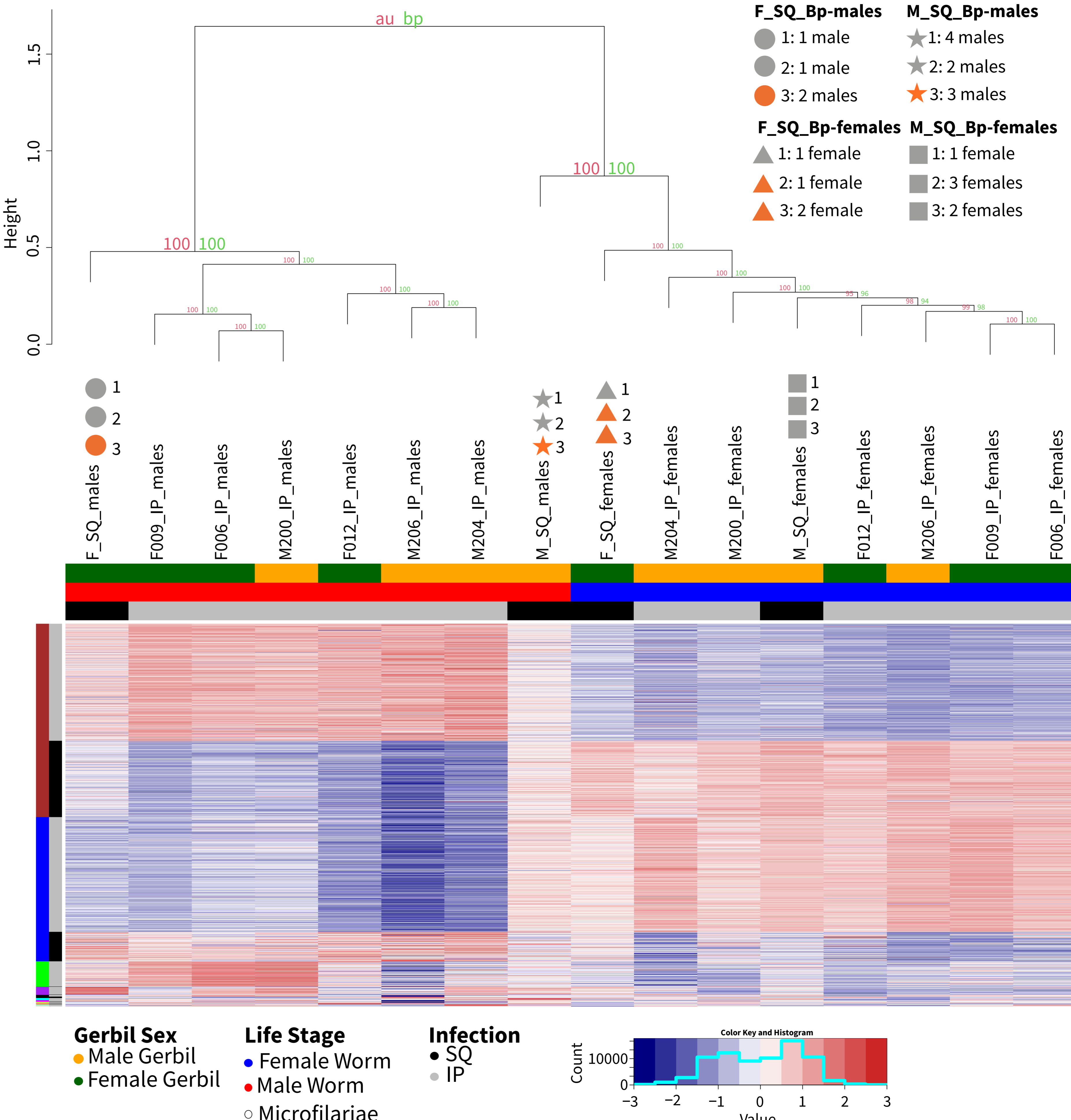

Supplement: jkag073_Supplementary_Data [file jkag073_Supplementary_Data.zip › Supplementary_Figure_5_G3-2026-406658.pdf]

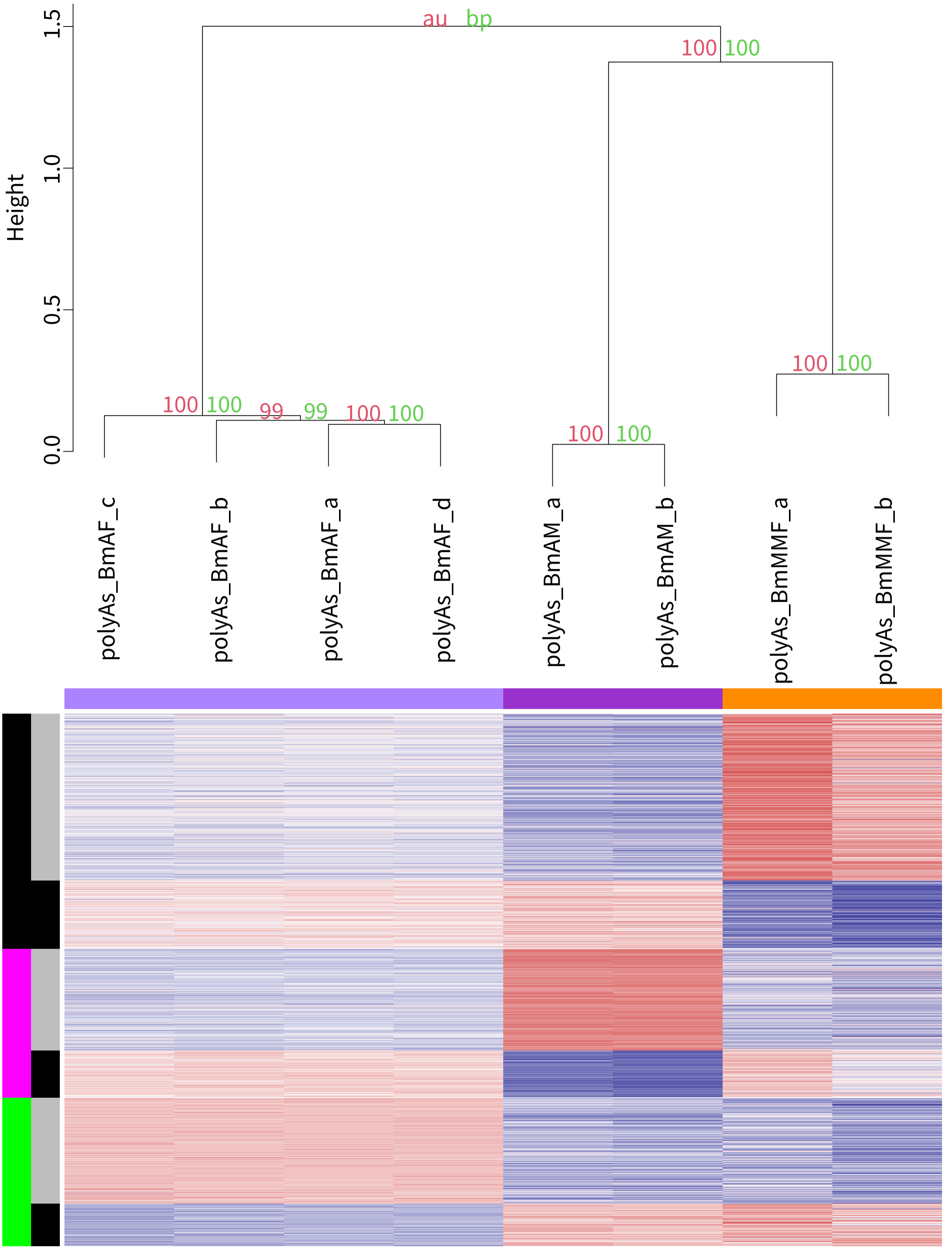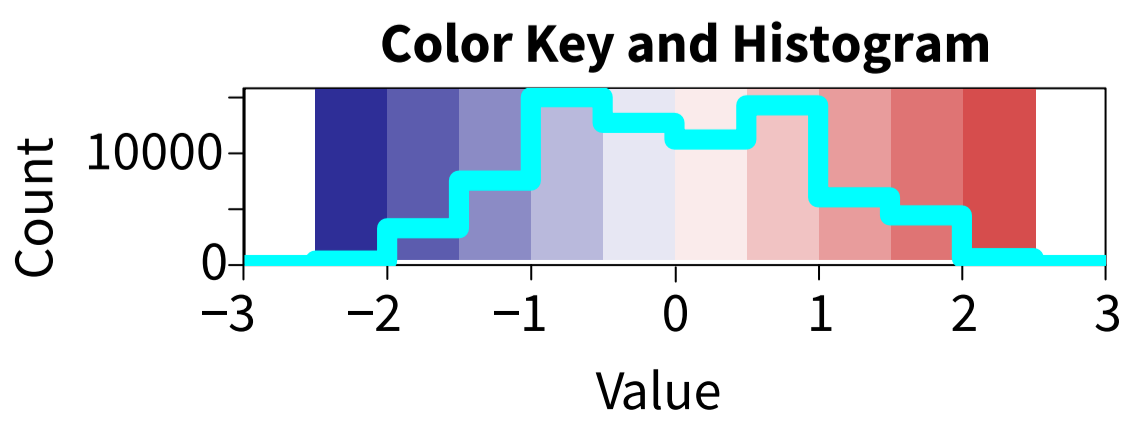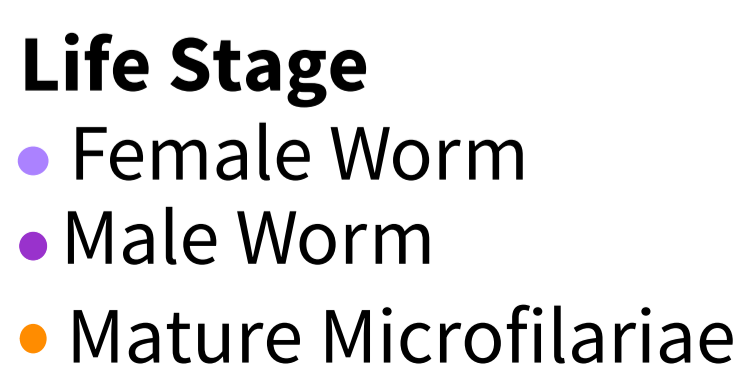

Supplement: jkag073_Supplementary_Data [file jkag073_Supplementary_Data.zip › Supplementary_Figure_6_G3-2026-406658.pdf]
